# The landscape of biomedical research funding in Brazil: a current overview

**DOI:** 10.1590/S1677-5538.IBJU.2024.9905

**Published:** 2024-03-18

**Authors:** Cristiano M. Gomes, Giovanni Marchini, Jose de Bessa, Gustavo Carvalhal, Marina P. R. Caldeira, Paulo Hilario Saldiva, Jose Eduardo Krieger, Fabiana Agena, Sabrina Reis, Candice Paschoal, Milena Froes, Miguel Srougi, William C. Nahas, Luciano A. Favorito

**Affiliations:** 1 Universidade de São Paulo Hospital das Clinicas da Faculdade de Medicina Departamento de Urologia São Paulo SP Brasil Departamento de Urologia, Hospital das Clinicas da Faculdade de Medicina da Universidade de São Paulo - FMUSP, São Paulo, SP, Brasil;; 2 Universidade Estadual de Feira de Santana Departamento de Cirurgia Feira de Santana BA Brasil Departamento de Cirurgia, Universidade Estadual de Feira de Santana - UEFS, Feira de Santana, BA, Brasil;; 3 Pontificia Universidade Catolica do Rio Grande do Sul Porto Alegre RS Brasil Pontificia Universidade Catolica do Rio Grande do Sul – PUC RS, Porto Alegre, RS, Brasil;; 4 Universidade de São Paulo Faculdade de Medicina Unidade de Apoio à Pesquisa e Inovação São Paulo SP Brasil Unidade de Apoio à Pesquisa e Inovação, Faculdade de Medicina da Universidade de São Paulo - FMUSP, São Paulo, SP, Brasil;; 5 Universidade de São Paulo Faculdade de Medicina Departamento de Patologia São Paulo SP Brasil Departamento de Patologia, Faculdade de Medicina da Universidade de São Paulo - FMUSP, São Paulo, SP, Brasil;; 6 Faculdade de Medicina da Universidade de São Paulo Hospital das Clínicas Instituto do Coração São Paulo SP Brasil Instituto do Coração, Hospital das Clínicas da Faculdade de Medicina da Universidade de São Paulo - FMUSP, São Paulo, SP, Brasil.; 7 Universidade de São Paulo Escola de Enfermagem São Paulo SP Brasil Escola de Enfermagem da Universidade de São Paulo, São Paulo, SP, Brasil;; 8 Instituto D'Or de Pesquisa e Ensino São Paulo SP Brasil Instituto D'Or de Pesquisa e Ensino, São Paulo, SP, Brasil;; 9 Universidade Estadual do Rio de Janeiro Unidade de Pesquisa Urogenital Rio de Janeiro RJ Brasil Unidade de Pesquisa Urogenital – Universidade Estadual do Rio de Janeiro - UERJ, Rio de Janeiro, RJ, Brasil

**Keywords:** Biomedical Research, Economics, Financing, Government, Brazil

## Abstract

**Objective:**

The objective of this narrative review is to discuss the current state of research funding in Brazil.

**Materials and Methods:**

This study is based on the most recent edition of the course Funding for Research and Innovation in the University of Sao Paulo School of Medicine which was a three-day course with 12 hours of instruction. The course brought together leading experts in the field to comprehensively discuss the current state of research funding in Brazil. Each speaker provided a presentation on a specific topic related to research funding. After the workshop, speakers assembled relevant topics in this manuscript.

**Results:**

collaborative research is critical for securing research funding. It optimizes proposal competitiveness, amplifies societal impact, and manages risks effectively. As such, fostering and supporting these collaborations is paramount for both researchers and funding agencies. To maintain the highest integrity in research, investigators involved in these collaborations must disclose any relationships that could potentially influence the outcomes or interpretation of their projects.

**Conclusions:**

In Brazil, the mainstay of research funding stems from public entities, with agencies such as CNPq, CAPES, and state bodies like FAPESP, FAPERJ, FAPEMIG and others at the forefront. Concurrently, industry funding offers viable pathways, especially through industry-sponsored studies, investigator-led projects, and collaborative initiatives. The Brazilian funding landscape is further enriched by innovative platforms, including crowdfunding and the contributions of institutions like the Serrapilheira Institute. Internationally, esteemed organizations such as the National Institutes of Health (NIH) and the Bill & Melinda Gates Foundation stand out as potential funders.

## INTRODUCTION

IScience funding is important to develop knowledge, technology, and foster innovation. Although it is universally accepted that it is essential to invest in biomedical research, funding in this field differs considerably among countries.

Total global investment in biomedical and health research was estimated at US$240 billion in 2009, equivalent to approximately US$300 billion in 2023 (1). Governments have been the main source for biomedical research funding throughout the World. The health gains arising from biomedical research are easy to demonstrate as they lead to new ways to prevent, diagnose, and treat illnesses, as seen in the recent development of effective vaccines and treatments for COVID-19. Moreover, biomedical research prepare world-class scientists and has the potential to bolster the economy and reduce the burden of illness (2, 3).

Funding is critical to maintaining research labs and active researchers, who often rely on research grants for their salaries or stipends. The NIH (National Institutes of Health – United States’ medical research agency) is the largest public funder of biomedical research in the World, providing research grants that support more than 300,000 researchers at more than 2,500 institutions in the United States. Other very important national agencies that fund biomedical research are the European Research Council (European Union), the Medical Research Council (United Kingdom), the Deutsche Forschungsgemeinschaft (Germany) and the National Natural Science Foundation of China.

In Brazil, research funding is provided through different systems and institutions, which are directly or indirectly linked to Brazilian ministries or federal agencies, such as the National Council for Scientific and Technological Development (CNPQ – Brazil), Coordination for the Improvement of Higher Education Personnel (CAPES), Financier of Studies and Projects (FINEP) and National Fund for Scientific and Technological Development (4). In addition, several state Foundations for Research Support provide funding, like FAPESP (Sao Paulo Research Foundation), Rio de Janeiro State Research Foundation (FAPERJ) and Minas Gerais State Research Foundation (FAPEMIG) (5, 6).

Significant constraints in research funding have been observed in many countries in the past few years and Brazil has been affected significantly (4, 5, 7). Because government research funding is limited, finding sources other than the government has become a top priority of several research groups (8–16).

The objective of this narrative review is to discuss the current state of research funding in Brazil.

## MATERIALS AND METHODS

Since many researchers and postgraduate students in the early phase of their careers do not have a proper understanding of the importance as well as of the process and opportunities for obtaining funding for scientific research in Brazil, we have developed a discipline entitled “Funding for Research and Innovation” in the University of Sao Paulo School of Medicine. This narrative review is based on the most recent edition of the course, which was a three-day course with 12 hours of instruction that was held in São Paulo, Brazil, in November 2022. The course brought together leading experts in the field to comprehensively discuss the current state of research funding in Brazil. Each speaker provided a presentation on a specific topic related to research funding. After the workshop, speakers assembled relevant topics in this manuscript.

The discipline focus on biomedical research funding in Brazil and our audience consists of post-graduation students (Master's or Doctoral students) from different backgrounds and with different levels of experience in biomedical research. The program was developed to address the domains: (1) Importance of funding in biomedical research, (2) Elements of a remarkable research project, (3) Opportunities with public funding Agencies for biomedical research in Brazil, (4) Industry funding for biomedical research, (5) Other funding opportunities; (6) Fundable items in a research project and (7) Step by step submission of a research project to a government funding institution. The instructional methods consist of alternating lectures and discussions as detailed in Table-1.

**Table 1:** Course programme.

Day	Time	Activity - Topic	Instructional method
1	08:00-08:15	Welcome and Introduction	
1	08:15-09:00	Fundraising Essentials: Understanding value, Ethics and financial Implications	Lecture and Q&A*
1	09:00-09:45	Navigating the CNPQ** Landscape: Opportunities & Unique Aspects	Lecture and Q&A
1	09:45-10:00	Break	Health
1	10:00-10:45	The Reviewer's perspective: Mastering Grant Success Elements and Priorities	Lecture and Q&A
1	10:45-11:30	A Guide to Grant Applications: Strategies to optimizing Success	Lecture and group discussions
1	11:30-12:00	Efficient Resource Management in sponsored Clinical Studies	Lecture and Q&A
**2**	**08:00-08:15**	**Opening of Day 2**	
2	08:15-09:00	The Role of the Private Sector and Philanthropy in Modern Research Funding	Lecture and Q&A
2	09:00-09:45	Innovative Research Funding: The Pivotal Role of FAPESP	Lecture and Q&A
2	09:45-10:00	Break	Health
2	10:00-10:40	Ensuring Quality: Addressing Bias in Funded Research and its Publication Impact	Journal club and group discussions
2	10:40-11:20	Crowdfunding: A Modern Frontier for Scientific Research Funding	Lecture and Q&A
2	11:20-12:00	Driving Innovation: A Look at Butantan's Financial model	Lecture and Q&A
**3**	**08:00-08:15**	**Opening of Day 3**	
3	08:15-09:00	Research at the University of São Paulo: Current funding profile and perspectives	Lecture and Q&A
3	09:00-09:45	The Pharmaceutical Industry's Role in funding Modern Research	Lecture and Q&A
3	09:45-10:00	Break	Health
3	10:00-10:30	The Editor's perspective: Perceptions of Industry-Funded Research in Indexed Journals	Lecture and group discussions
3	10:30-11:15	Research at Hospital Israelita Albert Einstein: Opportunities and challenges	Lecture and Q&A
3	11:15-12:00	Exploring Funding Prospects with International Agencies	Lecture and Q&A
3	12:00-12:05	Closing remarks	

## DISCUSSION

### Importance of obtaining funding for biomedical research

For researchers engaged in biomedical studies, obtaining funding is critical for the development of high quality research (17, 18). The economic resources provide capital for the acquisition of essential equipment, recruitment of skilled work force, and coverage of several research-related expenses. With funding, researchers can conduct higher quality studies that result in increased citations enhancing the overall scientific impact of their work (17, 19, 20).

In addition, securing funding may be important for career advancement. Success in grant applications is often perceived as an important quality and an indicator of potential for academic success in future (21). It not only proves the researcher's capacity to obtain financial resources, but also the ability to conceive and plan robust scientific investigations.

Beyond its impact on individual projects and careers, funding also facilitates broader collaborative studies (22, 23). It encourages the union of diverse academic disciplines, fostering a more comprehensive approach to research. Furthermore, securing funding amplifies a researcher's recognition and credibility within the scientific community. This increases the reach for disseminating their findings, providing better opportunities for sharing their work through publications and presentations. Essentially, funding does not only support the research projects, but also promotes the careers of the scientists behind the research, improving their reputation, and expanding their influence within the academy (24, 25).

For the institutions involved in scientific research, the acquisition of funding may also have a profound impact and may play an important role in ensuring their financial viability and supporting a wide array of institution-related costs (26). Research funds may provide the necessary resources to sustain and enhance the research infrastructure, including the acquisition of lab equipment, restoration of research facilities, and implementation of new research methodologies. Obtaining research funding continuously renders research institutions highly attractive to the most talented prospects within the scientific community, which often drives high quality research and ultimately elevates institution's prestige (27, 28).

Obtaining research funding also plays a pivotal role in preserving employment within the research sector and maintaining the operations of research facilities. Adequate funding ensures the continuation of scientific endeavors and sustains the livelihoods of many within the research realm.

### Characteristics of a remarkable research project

Undertaking the preparation and submission of a biomedical research grant application is a significant commitment. This highly competitive task can be threatened by insufficient planning, inadequate preparation, disorganization, and uninspiring presentation. The proponent must be sure to allow sufficient time to plan, organize, and complete a grant application that stands out in the peer review process.

This section provides tips and strategies for planning and organizing your application. It is important to collaborate closely with your institution's grant support office or the equivalent department that oversees sponsored programs, to understand the internal protocols for submitting an application. The advice provided here is primarily oriented towards Research Project Grants. The tips and guidance provided in this document are not intended to supersede an organization's internal guidelines, specific advice from program or grants management staff, or instructions from various application guides. The study proposal must rise above plenty of submissions, demonstrating innovative thinking and scientific rigor. In addition, having the potential to impact public health is certainly an advantage. Considering this, several key characteristics define a remarkable research project suitable for funding:

1. Significance and Innovation: The project must address a significant question or issue whether it pertains to a clinical problem or explores fundamental physiological or pathophysiological topics, using a novel approach. The innovative aspect could stem from the problem itself, the methodology, or the anticipated results. The proposal should make clear the urgency and relevance of the research question and how the innovative approach can provide groundbreaking insights (8, 29).
2. Clear and feasible objectives: A well-defined objective that is based on a testable hypothesis is crucial. It should be relevant, specific, measurable and achievable. It is equally important to ensure that the objectives can be feasibly achieved within the proposed timeframe and budget. It has been shown that completion of a pilot or feasibility study is a strong predictor of success in obtaining funding (30).
3. Scientific and methodological rigor: The project should have a robust and reproducible methodology. This includes clearly defined population and procedures, control measures, and data analyses plans. Rigor and transparency in methodology not only increase the credibility of the project but also allow for replication and validation of the results by other researchers.
4. Interdisciplinarity and collaborative approach: Involving researchers with different backgrounds and expertise may improve the quality of a research project (29, 31). Increasingly, biomedical research is becoming interdisciplinary, involving experts from different fields such as biology, epidemiology, medicine, bioinformatics, and more. Such collaborative efforts can help address complex research questions from multiple angles and potentially yield more impactful results (22).
5. Strong research team: The expertise and experience of the research team is essential (31). A diverse team, where each member brings unique skills and knowledge, will add credibility to the project. Government funding agencies value the track record of the Principal Investigator (PI) and team members in carrying out successful research. In addition, working in a well-recognized research institution seems to be a positive characteristic for increasing the odds of getting a research proposal funded (26). Researchers in the beginning of their career may improve their chances of getting funded by collaborating with accomplished investigators (23, 30, 32).
6. Ethical aspects: A high-quality research proposal must clearly address ethical considerations, including patient consent, privacy, and data security. It should demonstrate that the benefits of the research outweigh potential risks to the participants.
7. Dissemination and translation of results: The project should have a plan for disseminating research results, translating the outcomes into policy or practice, or commercialization of the product if relevant. A good dissemination plan increases the potential impact of the research.
8. Patient and public involvement: Research that involves the public or patients in its design and execution can provide real-world context and relevance. It shows the agency that the project is not only theoretically sound but also practical, and that it is likely to make a tangible difference to the intended beneficiaries.

In summary, a remarkable biomedical research project suitable for government funding is characterized by its significance, novelty, feasibility, rigor, collaborative nature, strong team, ethical soundness, and potential for impact. Preparing a project with these features increases the likelihood of securing funding from government agencies (33).

### Fundable items in a research project

Generally, government entities that fund scientific research follow comparable guidelines for providing financial support and permit the incorporation of similar expenditure items. Expense items are classified as Capital (permanent material acquired) and Operating Expenses (consumables, third-party services, travel, and daily allowances).

Permanent material includes the purchase of equipment, furniture, computers, machinery, bibliographic material, vehicles, renovations, or installations.

Operating Expenses include consumables, payment for clinical analyses, service providers, air/land travel, and daily allowances.

It is imperative that researchers justify each item requested, based on the proposed objectives and expected results of the study.

In addition to the expense items that are inherent to the study proposal, financing agencies may provide additional resources intended for unforeseen expenses directly related to the projects.

At FAPESP, the “Technical Reserve” is an integral part of most study grants and is divided into ‘Complementary Benefit’ and ‘Direct Infrastructure Costs of the Project.’ The ‘Complementary Benefit’ is primarily intended to cover expenses with participation in scientific or technological meetings, either nationally or internationally. However, if the resource is not used for this purpose, it can cover unforeseen Capital and Operating Expenses in the project. The ‘Direct Infrastructure Costs of the Project’ is exclusively intended to cover costs/services related to Capital and Operating Expenses NOT initially foreseen in the project.

Research grants from government funding agencies in Brazil do not cover researchers’ salaries or stipends. It is expected that these will be provided by the institution to which the principal investigator is affiliated. Associated researchers involved in the study may receive a scholarship that can be included in the study proposal or requested from the funding agency separately, regardless of the specific project.

With respect to studies funded by the pharmaceutical industry, the budget can include all items necessary for conducting the research, including the payment of the researchers involved.

Studies financed by philanthropic or crowdfunding initiatives usually have more flexible resource allocation rules due to a lack of strict oversight on resource utilization. This does not imply unrestricted use of funds, but rather that these projects have less stringent accounting requirements, and their success is generally assessed based on the achievement of initially proposed objectives.

### Public funding for biomedical research in Brazil

Brazilian investment in research and development (R&D) is approximately 1.3% of the GDP, according to Unesco (34). This figure is lower than that of top-performing countries such as the USA, which dedicates 2.7% of its GDP to R&D. In countries with substantial R&D investments, the private sector often contributes a significant share, sometimes up to 70%. In Brazil, around 45% of R&D investment originates from the private sector, while the government provides the remainder (35).

Brazil's public funding system for biomedical research has faced challenges over the years. The system, encompassing several governmental agencies, experiences fluctuations and is influenced by the priorities of the current government.

Research funding is provided through different systems and institutions, which are directly or indirectly linked to Brazilian ministries or federal agencies. These include CNPQ *(Conselho Nacional de Desenvolvimento Científico e Tecnológico)*, CAPES *(Coordenação de Aperfeiçoamento de Pessoal de Nível Superior)*, FINEP *(Financiadora de Estudos e Projetos)*, and FNDTC *(Fundo Nacional de Desenvolvimento Científico e Tecnológico)* (4).

CNPQ is a federal entity that stands out in the role of promoting scientific and technological research in Brazil. With a mission to foster scientific and technological research, CNPQ has consistently supported researchers in various biomedical fields, including but not limited to medicine, biology, pharmacology and physiotherapy. One of its hallmark initiatives is the provision of scholarships to a diverse group, ranging from budding students to seasoned researchers. This not only aids in the individual growth of recipients but also ensures a constant stream of talent into the field of biomedical research. Through its consistent efforts and initiatives, CNPQ has established its position as a pillar in fostering new biomedical researchers in Brazil.

CAPES primarily funds scholarships at the postgraduate levels (master's, doctorate, and post-doctorate), with limited opportunities for project funding.

The Brazilian Innovation Agency (FINEP - *Financiadora de Estudos e Projetos*) represents the government's commitment to promoting technological and innovative projects, including those within the biomedical field (34). Often, FINEP partners with other agencies and the private sector to fund research with commercial potential. The Brazilian Ministry of Health is also instrumental in sponsoring biomedical research, predominantly those linked to pressing public health concerns. Its efforts cover initiatives to enhance healthcare infrastructure and address critical health challenges.

Several state Foundations for Research Support finance research (5, 6) They often collaborate with federal entities and the private sector to sponsor state-level biomedical research. This manuscript focus in the agencies of São Paulo and Rio de Janeiro, the two states with the most substantial research budgets.

FAPESP is a premier Brazilian research agency supporting projects across various disciplines, including biomedical sciences. Researchers should monitor FAPESP's website for the latest funding opportunities. The “Regular project” grant provides up to R$ 300,000.00 for individual principal investigators, while the “Thematic project” offers extensive funding for specific biomedical research led by collaborative teams of experienced scientists. In addition to funding projects, FAPESP offers scholarships from scientific initiation to post-doctoral levels and has special programs to nurture early career researchers, emphasizing research excellence (38).

FAPERJ supports scientific advancement in Rio de Janeiro. Aimed at boosting socioeconomic development through research, FAPERJ offers diverse funding options, benefiting both emerging and established researchers. The agency emphasizes cutting-edge areas like artificial intelligence, biotechnology, renewable energy, and climate change.

The various federal and state agencies typically advertise grant support opportunities in their respective websites. It's essential to seek out opportunities aligned with the applicant's research interests or apply through generic parent announcements tailored for a broad range of topics.

### Industry funding for biomedical research:

Industry funding plays a significant role in advancing biomedical research (36–39). In this session, we will explore three aspects of industry funding in biomedical research: clinical industry-initiated studies, investigator-initiated studies, and collaborative research.

Industry funding through *clinical industry-initiated studies* is essential for evaluating the safety and efficacy of new drugs, medical devices, and therapies in similar or innovative approaches of the pivotal clinical trials (38, 40). Pharmaceutical and medical device companies typically sponsor these studies to expand the knowledge about their products or therapeutic areas. One advantage of industry funding for clinical studies is the substantial financial resources it provides. This funding enables researchers to conduct large-scale trials, recruit participants, and collect comprehensive data, enhancing the statistical power and generalizability of study findings (41, 42). Industry funding also allows for the utilization of specialized equipment, technology, and expertise that may not be readily available in academic or public research settings.

To overcome potential conflicts of interest, researchers must ensure the independence and integrity of the study design, data collection, and analysis to maintain scientific rigor and objectivity. To address these concerns, regulatory bodies and research institutions have implemented transparency and disclosure requirements and the collaboration between academic researchers and industry partners can also help mitigate potential conflicts of interest and ensure the research is conducted in an ethical manner (37, 39).

In addition to industry-sponsored studies, *investigator-initiated studies* that receive industry funding play a crucial role in biomedical research. These studies are initiated and led by independent researchers, who propose research projects aligned with their scientific interests and expertise. These studies offer researchers the opportunity to explore novel hypotheses and investigate innovative approaches. Funding provides financial support for research materials, personnel, data collection, and analysis, enhancing the feasibility and quality of the study. By investing in independent research projects, industries demonstrate their commitment to scientific progress and patient welfare beyond their commercial interests. This can enhance public trust in the industry and strengthen the collaboration between academia and the private sector. As in the industry-initiated studies, researchers must ensure that the funding source does not compromise the study's design, data analysis, or interpretation of results. Transparency in disclosing funding sources and potential conflicts of interest is essential to assure the scientific rigor.

Academics can provide unbiased expertise and access to patient populations, while industry partners can offer financial support, specialized knowledge, and access to resources. Such collaborations may accelerate the translation of scientific discoveries into clinical applications and contribute to improving patient care.

*Collaborative research* is a format of sponsored studies practiced by some industries. This alternative allows for the true collaboration between industry and researchers to plan and execute research studies. This partnership is based on similar expertise and research interests from both parts. Leveraging scientific acumen from industry and academia in that particular area of knowledge can strengthen study planning and expedite execution, while optimizing budget. In this type of funding all steps of the research are agreed between both parts.

Those are general approaches to research funded by industry and may vary among the different companies. Generally, a common practice among them is to support research in the therapeutic areas they act on. Requests for funding may be enduring or via specific calls for application. Thus, it is important for researchers to understand the synergy between their field of research and the area of interest of the different companies to explore opportunities. Information can be obtained in companies' websites and with local Medical Affairs teams.

Finally, industry-sponsored studies often result in unused budget provisions. These surplus funds can be redirected to areas of research that may be underfunded or neglected, addressing unmet medical needs. Alternatively, it can be used for the maintenance or renewal of the institution's infrastructure.

### Quality of studies funded by industry:

Industry funding has attracted significant criticism due to its perceived influence on the research agenda and its potential impact on the quality of resulting publications. One of the key concerns raised by critics is the potential for industry sponsorship to shape the direction and focus of research (43). In many cases, industry-funded studies tend to align with the commercial interests of the sponsoring companies, which may prioritize research that supports their products or services rather than pursuing unbiased scientific inquiry (44).

Critics argue that this influence can be seen in various aspects of the research process. For instance, industry sponsorship has been known to impact the selection of research questions (45). Funding from pharmaceutical companies, for example, may result in an overemphasis on drug development and clinical trials for specific medications, while neglecting other important areas of research.

Moreover, industry funding can influence the study design and methodology employed in research projects. Sponsoring companies may exert pressure to use certain methodologies that are more likely to yield favorable results for their products or to exclude certain control groups that could potentially reveal adverse effects (44).

Another concern lies in the interpretation and dissemination of research findings. Critics argue that industry-funded studies tend to present results in a manner that favors the sponsoring company's interests, potentially downplaying negative findings or exaggerating positive ones (44, 45). This selective reporting can skew the overall evidence base and hinder a comprehensive understanding of the subject matter. Reviews support this notion that industry-funded studies often report outcomes more favorable to the sponsor than those not financed by the industry (43–45).

Critics also highlight instances where conflicts of interest are not properly disclosed, leading to a lack of transparency in the research process. Failure to disclose financial ties between researchers and industry sponsors can undermine the credibility and objectivity of the research, raising doubts about the reliability of the findings (46).

While concerns about bias exist, industry-funded studies are often high-quality multicenter studies, with a large sample size that supports generalizability. It would be ideal to use such robust studies to obtain strong and meaningful scientific results.

Influencing the industry's research agenda may be challenging, but strategies can be employed to lessen bias within industry-funded research. Adherence to ethical principles must be rigorous across all stages of research to sustain its integrity and reliability. Furthermore, peer review has a major role in assuring research quality. It serves as an external control for research methodology and findings and is key to ascertain their validity. The association of rigorous scrutiny provided by peer review and the transparency and collaboration fostered by open science creates a powerful framework for ensuring robust, credible, and accessible scientific research.

### Other funding opportunities in Brazil

In recent years, the global landscape of research funding has undergone a significant transformation. A number of countries worldwide, including Brazil, have encountered notable constraints in obtaining government research funding (5, 7, 36, 47). This phenomenon is particularly pronounced in the field of biomedical research, typically characterized by substantial financial requirements due to the high costs of experimentation and clinical trials. This situation has brought the search for alternative sources of funding to the forefront of discussion, with the exploration of new strategies for overcoming these challenges.(4, 10, 14, 36, 48, 49).

One such innovative approach that has emerged in the landscape of research funding is *Crowdfunding*, that has been used to support research studies in many countries (50–52). It engages large groups of people who make small contributions to support a research study, providing a method for researchers to engage with the public (50, 53). Crowdfunding provides a way for communities and stakeholders to invest in locally relevant topics and directly contribute to scientific research.

Based on the principles of community contribution and democratization of support, crowdfunding empowers researchers to bypass the limitations imposed by traditional funding channels and directly appeal to the public for financial support (10–12). Through the use of online platforms, researchers are enabled to present their projects to a broad, diverse audience, thereby reaching out to individuals who are passionate about specific medical areas and/or interested in the advancement of medicine.

This strategy for funding presents an opportunity for researchers who may have found difficulties in obtaining grants through conventional means to nonetheless pursue their research goals (12). Secondly, by engaging the general public in the research process, it favors a sense of community involvement in scientific advancements. This not only generates financial support but also improves public understanding and appreciation of science (54, 55).

Crowdfunding also allows for a distinct degree of flexibility, which traditional funding sources often lack. Unlike these conventional sources, which typically have rigid requirements and timelines, crowdfunding allows researchers to set their own goals and adapt their projects according to evolving circumstances and findings. This adaptability proves particularly beneficial for exploratory or innovative research projects that may be less appropriate for standard funding models.

Moreover, crowdfunding serves as a potent tool for networking and increasing exposure. Crowdfunding campaigns offer researchers a platform to showcase their work, gaining visibility within the medical community and beyond. Through social media and online platforms, researchers can attract attention from potential collaborators, industry partners, and even traditional funding agencies. This increased exposure can facilitate the building of a network of supporters, creating opportunities for future collaborations and additional funding avenues.

In Brazil, the *Serrapilheira Institute* serves as an example of a non-governmental organization contributing substantially to the funding of scientific research. The Institute, with its mission of supporting innovative and high-impact projects, offers several opportunities for medical researchers. By providing grant programs designed to support medical research initiatives, it allows scientists to explore novel and risky research projects. From 2018 to August/2022 it invested R$ 51.868.416,42, distributed to 152 different research projects (56).

The Institute encourages collaborative research initiatives and supports events like workshops and conferences. In addition, it offers opportunities for professional development and networking. Additionally, recognizing the critical role of effective science communication, the Institute has implemented communication and outreach programs that assist researchers in disseminating their findings to a broader audience.

The shift in the landscape of research funding presents both challenges and opportunities for the world of biomedical research. While traditional government funding remains an essential component of the research ecosystem, the growth of alternative funding avenues, including crowdfunding and non-governmental organizations, provide a more diversified and democratic model. As researchers continue to navigate these changes and explore diverse funding opportunities, it's clear that these alternative sources will play an increasingly important role in driving scientific progress and fostering a vibrant and robust research community in Brazil and beyond.

## FUNDING FROM FOREIGN INSTITUTIONS

### National Institutes of Health (NIH)

The National Institutes of Health (NIH) is a leading global medical research agency that provides a wide range of funding opportunities to support biomedical research. The NIH categorizes its funding based on various research, conditions, and disease categories. These categories are based on grants, contracts, and other funding mechanisms used across the NIH (57). Additionally, disease burden data published by the National Center for Health Statistics (NCHS) at the Centers for Disease Control & Prevention (CDC) are also reported alongside the budgeting categories.

Foreign researchers can apply for NIH grants through a similar process as domestic researchers. The application process generally involves identifying the appropriate funding opportunity, preparing a detailed research proposal, and submitting it through the NIH's electronic submission system. In general, foreign institutions and international organizations, including public or private non-profit or for-profit organizations, are eligible to apply for research project grants (58). However, some NIH programs/mechanisms have a citizenship requirement. Any citizenship requirement will be stated in the program announcement (PA) or request for applications (RFA).

Foreign institutions and international organizations are not eligible to apply for Kirschstein-NRSA institutional research training grants, program project grants, center grants, resource grants, SBIR/STTR grants, or construction grants. However, some activity codes, such as program project grants (P01), may support projects awarded to a domestic institution with a foreign component. Foreign applications must be presented to the NIAID advisory Council as a special issue to obtain approval. A foreign component cannot be added to a grant without obtaining prior approval of the grants management officer (GMO) (58).

### Bill & Melinda Gates Foundation

The Bill & Melinda Gates Foundation is a leading global philanthropic organization that provides a wide range of funding opportunities to support research in various fields (59). The foundation awards the majority of its grants to U.S. organizations and other tax-exempt organizations identified by their staff. However, they also welcome applications from international researchers. The application process for foreign researchers is similar to that of domestic researchers. The first step in the application process is identifying the appropriate funding opportunity. The foundation does not make grants outside its funding priorities. In general, they directly invite proposals by contacting organizations. However, they do occasionally award grants through published Requests for Proposals (RFPs). Therefore, it is crucial for researchers to keep an eye on the list of current RFPs.

Once an appropriate funding opportunity has been identified, the next step is preparing a detailed research proposal. The proposal should include a comprehensive narrative of the proposed research, a detailed budget, and sometimes a results framework and tracker. After the proposal has been prepared, it should be submitted through the foundation's electronic submission system. Once a grant is approved, the grantee will typically rely on the investment document progress narrative section and grant budget to report formally on progress, challenges, and financial status. It's important to note that the foundation does not make grants directly to individuals except in specific circumstances as noted on certain grant applications. Also, they make grants to organizations directly rather than through individual fundraising activities (59).

In conclusion, while the process of applying for resources at the Bill & Melinda Gates Foundation as a Brazilian researcher involves several steps and requires careful preparation, it is certainly feasible. By staying informed about current RFPs, preparing a thorough research proposal, and following the foundation's application guidelines, Brazilian researchers can successfully navigate this process.

### Impact of collaboration for improving funding

Collaborative research plays a pivotal role in advancing biomedical knowledge and fostering innovation. Within the context of biomedical research funding, promoting collaborations among academic institutions, industry partners, and governmental agencies is essential (60).

Collaborative research brings together diverse expertise and resources from multiple stakeholders (23). By combining knowledge and capabilities, researchers can tackle complex questions and interdisciplinary challenges (61, 62). It facilitates access to specialized medical equipment, state-of-the-art facilities, and extensive medical databases that may not be readily available to individual researchers or institutions. This amplification of expertise and resources enhances the overall appeal and competitiveness of research proposals (63).

When academia collaborates with industry partners, the research becomes more closely aligned with healthcare market needs, ensuring its clinical relevance and potential for commercialization (64). This alignment enhances the chances of securing funding from industry sponsors. The active involvement of healthcare providers and stakeholders throughout the research process also augments the likelihood of achieving real-world impact, thereby increasing the prospects for funding support.

Collaboration also distributes inherent research risks. There is a significant challenge in moving from promising scientific observations to the creation of effective therapies. This process is not only expensive, but many times frustrating since most therapeutic developments stumble at the preclinical stage. By sharing responsibilities and resources, the burden on individual medical researchers or institutions is significantly reduced (65). This is especially true for therapies for niche diseases, that might serve only specific markets. The pharmaceutical industry often shies away from early-stage programs, especially for rare or ‘orphan’ diseases. Recognizing this gap, federal agencies have developed programs to spark innovation and lower the hurdles for new therapeutic introductions (66).

Beyond resources, collaborations facilitate knowledge exchange, offering researchers fresh perspectives and innovative methodologies. It also helps establishing robust collaborative networks, even on an international scale, enabling access to international funding opportunities and global medical research networks.

In summary, collaborative research is critical for securing research funding. It optimizes proposal competitiveness, amplifies societal impact, and manages risks effectively. As such, fostering and supporting these collaborations is paramount for both researchers and funding agencies. To maintain the highest integrity in research, investigators involved in these collaborations must disclose any relationships that could potentially influence the outcomes or interpretation of their projects.

## CONCLUSION

Biomedical research thrives with adequate funding, a cornerstone essential for driving innovations and advancing healthcare. A standout research project poised for funding typically showcases clear objectives, rigorous methodology, and the potential for a marked impact. Collaborations, involving both local and international researchers, not only bolster funding opportunities but also amplify the potential results and significance of the research.

In Brazil, the mainstay of research funding stems from public entities, with agencies such as CNPq, CAPES, and state bodies like FAPESP, FAPERJ, FAPEMIG and others at the forefront. Concurrently, industry funding offers viable pathways, especially through industry-sponsored studies, investigator-led projects, and collaborative initiatives. The Brazilian funding landscape is further enriched by innovative platforms, including crowdfunding and the contributions of institutions like the Serrapilheira Institute. Internationally, esteemed organizations such as the National Institutes of Health (NIH) and the Bill & Melinda Gates Foundation stand out as potential funders.
